# Investigation on Anti-Fuel Erosion Performance of Sasobit/SBS-Modified Asphalt and Its Mixtures

**DOI:** 10.3390/ma17123016

**Published:** 2024-06-19

**Authors:** Yongkang Wu, Meizhu Chen, Qi Jiang, Jianwei Zhang, Yansong Fan, Jun He

**Affiliations:** School of Materials Science and Engineering, Wuhan University of Technology, Wuhan 430070, China; wuyongkang@whut.edu.cn (Y.W.); jiang7001@whut.edu.cn (Q.J.); zhangjianwei@whut.edu.cn (J.Z.); fys@whut.edu.cn (Y.F.); 331290@whut.edu.cn (J.H.)

**Keywords:** asphalt pavement, fuel erosion, Sasobit, anti-fuel erosion performance

## Abstract

The fuel leakage of fuel vehicles will exacerbate the occurrence of distresses on asphalt pavements, including peeling, chipping and potholes, especially under the synergistic effect of traffic load and environment. In this research, Sasobit, which is commonly used as a warm agent in asphalt, is selected as the anti-fuel erosion agent and incorporated into SBS-modified asphalt and its mixtures. Diesel and gasoline are selected as the fuel erosion media. Sasobit/SBS-modified asphalt binder and its mixtures are investigated for fuel erosion. The rheological properties of bitumen and the mechanical properties of asphalt mixtures are assessed. The experimental findings show that the dissolution velocity of SBS-modified asphalt with 3% Sasobit is 0.2%/min for diesel erosion, while it is 1.7%/min for gasoline erosion, lower than the control sample without Sasobit. Meanwhile, the rutting factor of Sasobit/SBS-modified asphalt decreases less than that of the control sample without Sasobit. Furthermore, the mass loss ratio after the Cantabro test of Sasobit/SBS-modified asphalt mixtures is 1.2% for diesel erosion, while it is 6.8% for gasoline erosion, lower than that of the control sample without Sasobit. The results of the mechanical properties for asphalt mixtures demonstrate that Sasobit can enhance the anti-fuel erosion performance. Moreover, the research results of the Sasobit modification mechanism show that Sasobit can form a microcrystalline structure in SBS-modified asphalt, which subsequently improves the anti-fuel of asphalt and its mixtures. This research provides a reference for anti-fuel erosion assessment methods and solutions to improve the anti-fuel erosion of asphalt pavement.

## 1. Introduction

**Figure 1:**
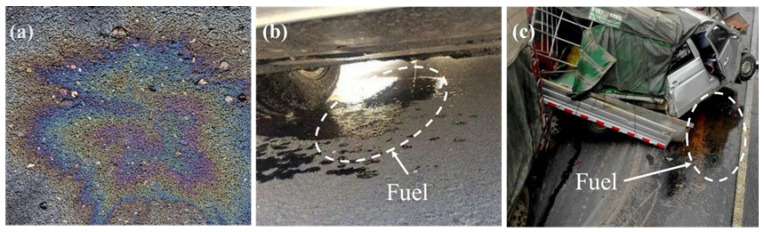
Fuel pollution of asphalt pavements: (**a**) fuel film of asphalt pavement, (**b**) fuel leakage due to vehicle breakdown and (**c**) fuel leakage due to traffic accidents.

It is very common for fuel leakage to occur frequently on asphalt pavements due to car breakdowns and traffic accidents, especially at gas stations, parking lots and airports [1,2], as shown in Figure 1. Moreover, fuels such as diesel are commonly used as isolation agents during the paving process of asphalt mixtures. It is common knowledge that asphalt and fuels such as diesel and gasoline are easily soluble in fuel due to their similar chemical compositions. Consequently, fuel erosion results in adhesion failure between asphalt and aggregate, exacerbating the occurrence of distresses on asphalt pavement, including peeling, chipping and potholes, especially under the synergistic effect of traffic load and the environment [3]. With the increasing number of cars and the widespread use of asphalt pavements, the harm of fuel leakage on asphalt mixtures will intensify. Therefore, it is meaningful to investigate the anti-fuel erosion performance assessment method and measures of asphalt pavements.

At present, the assessment methods of the anti-fuel erosion of asphalt mixtures are mainly based on the Marshall stability (MS) test [4,5], splitting tensile strength test [4,6], rutting test [7] and freeze–thaw splitting test [8]. Moreover, the mass loss ratio of asphalt mixtures after fuel immersion can quickly and intuitively evaluate the anti-fuel erosion performances of asphalt mixtures, and it is widely used [9,10,11]. The adhesion failure between bitumen and aggregate is the main cause of fuel erosion [12]; however, the mechanical tests do not focus on this main mechanism.

Scholars have conducted research to enhance the anti-fuel erosion abilities of asphalt pavements. The design of a dense asphalt pavement with small porosity can effectively reduce fuel erosion damage [6]. Furthermore, the sealants and anti-fuel erosion agents have been investigated. Sealants such as silicone resins [13], styrene-acrylic resins and vinyl resins [14] effectively segregate asphalt and fuel and exhibit good anti-fuel erosion performance. However, the sealant has many disadvantages, including the use of expensive materials, complex processes and a sharp decline in anti-fuel erosion performance when the sealant coating cracks. A better strategy appears to be applying proper asphalt modifiers to improve the anti-fuel erosion performance of asphalt. At present, polymers [15] and rubber crumbles [16] can serve as anti-fuel erosion agents, including some commercially available anti-fuel erosion products with undisclosed compositions [17]. Filippo et al. [18] added the same amounts of polymer materials, recycled rubber chips and synthetic waxes to the matrix asphalt and tested the anti-fuel erosion performances of these asphalts. The results show that the asphalts with synthetic waxes have a fairly high anti-fuel erosion performance. It is speculated that wax may form a physical barrier in the asphalt binder, in some way hindering fuel dissolution.

Sasobit has been widely used as a warm mix agent in asphalt mixtures and, consequently, to reduce harmful emissions and save energy [19,20,21]. The main components of Sasobit are n-alkanes and iso-alkanes. When Sasobit is added to the asphalt, Sasobit adsorbs some saturated components in the asphalt and crystallizes together to form a microcrystalline structure. This improves the high-temperature stability of asphalt, and compared with paraffin, the microcrystalline formed using Sasobit has less effect on the low-temperature performance of asphalt. However, there is little research in the literature on Sasobit’s use as an anti-fuel erosion agent in asphalt mixtures. Fuel leakage is random, and it is necessary to improve the anti-fuel ability of asphalt pavement as a whole. Considering the economic cost, Sasobit is used as an anti-fuel erosion agent to study its improvement effect on the anti-fuel erosion of asphalt pavement while still having a warm mix effect.

**Figure 2:**
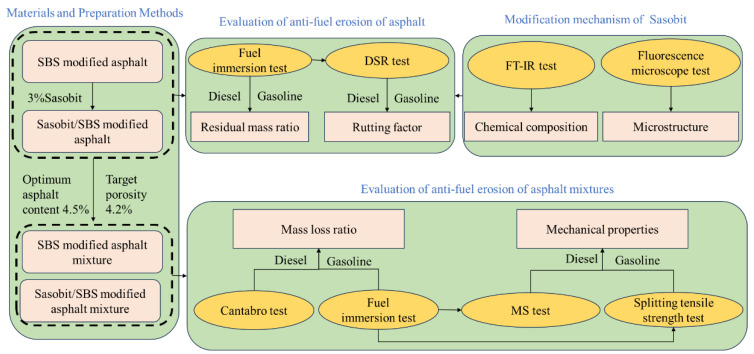
The experimental flow chart of this research.

The primary objective of this research is to systematically explore the anti-fuel erosion performance of Sasobit/SBS-modified asphalt and its mixtures, considering that SBS-modified asphalt is commonly applied in pavements. And the concrete experimental plan is demonstrated in Figure 2. The improvement in the anti-fuel erosion performance of SBS-modified asphalt generated by Sasobit is evaluated via the fuel immersion test. The dynamic shear rheometer (DSR) test is carried out on the asphalt after fuel immersion to explore the influence of fuel erosion on the rheological properties of asphalt. In addition, the modification mechanism of Sasobit in SBS-modified asphalt is analyzed via Fourier-transform infrared spectroscopy and fluorescence microscopy. The improvements caused by Sasobit in the anti-fuel performances of SBS-modified asphalt mixtures are evaluated via the fuel immersion test. In order to explore the influence of fuel erosion on the mechanical properties of asphalt mixtures, the asphalt mixtures after fuel immersion are subjected to the Cantabro test, Marshall stability (MS) test and splitting tensile test.

## 2. Materials and Methods

### 2.1. Raw Materials

SBS-modified asphalt is the most extensively utilized material for highways in China, making it a representative choice for this research. The I-D SBS-modified asphalt used in this research comes from a company in Hubei, China, with an SBS content of 4.7%. The properties of SBS-modified asphalt were assessed based on the standard test method for asphalt in China [22] and are represented in Table 1. 

Sasobit, in the form of white granules, is a polyolefin compound with a melting temperature of about 100 °C. Its performance indicators are presented in Table 2. When Sasobit is added to asphalt, it melts with the asphalt binder above the temperatures of 100 °C, making the asphalt binder less viscous. In this research, Sasobit was incorporated to SBS-modified asphalt and its mixtures as an anti-fuel erosion agent. 

**Figure 3:**
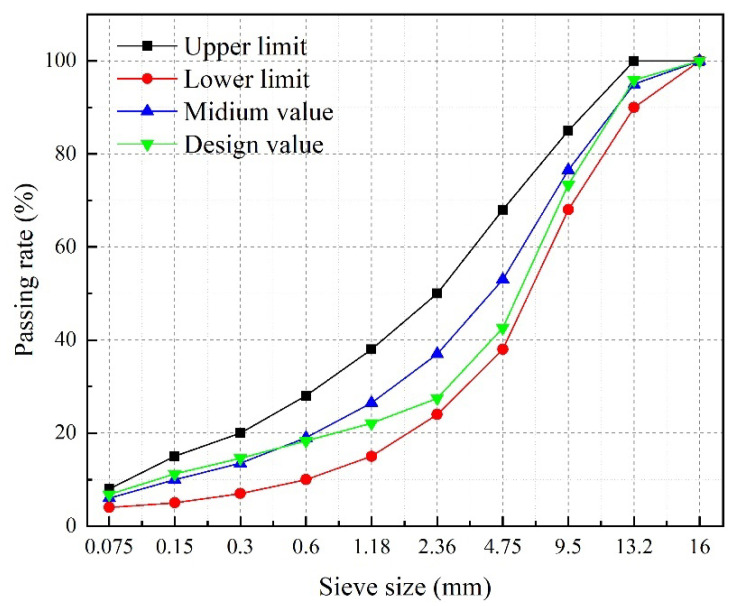
Aggregate gradation for AC-13 asphalt mixtures.

Basalt and limestone were selected as aggregate and filler, respectively. These materials meet the requirements of specifications in China [23]. AC-13 gradation is used in asphalt mixtures, as it is a typical aggregate gradation of asphalt pavement. The aggregate gradation used in this research is illustrated in Figure 3. 

### 2.2. Preparation of Sasobit/SBS-Modified Asphalt and Its Mixtures 

A shear mixer is used to homogenize Sasobit and SBS-modified asphalt, forming Sasobit/SBS-modified asphalt. The mixing parameters include a temperature of about 150–160 °C, a speed of 1500–2000 rpm and a mixing time of 20 min [24,25]. Sasobit is recommended to be added at a ratio of 0.8% to 3% by mass of asphalt [26]. At the same time, a dosage of 3% is the optimal amount of Sasobit usable as a warm mix agent for SBS-modified asphalt [19]. In this research, Sasobit is investigated as both an anti-fuel erosion agent and a warm mixing additive for asphalt mixtures. Therefore, a 3% dosage of Sasobit is incorporated into SBS-modified asphalt. The main properties of Sasobit/SBS-modified asphalt are shown in Table 3. 

Comparing the properties of two types of asphalt in Table 1 and Table 3, it is evident that the penetration is lower while the softening point is higher for Sasobit/SBS-modified asphalt, indicating that Sasobit can improve the high-temperature stability of asphalt. Meanwhile, Sasobit reduces the viscosity of asphalt binder at 135 °C, consequently lowering the mixing and compaction temperatures of asphalt mixtures [20]. Meanwhile, Sasobit reduces the ductility of SBS-modified asphalt and has a slight negative effect on the low-temperature properties of asphalt [27]. 

The Marshall design methodology was utilized to optimize the optimum asphalt content of AC-13 asphalt mixtures according to the JTG F40-2004 standard in China [23]. The optimum asphalt content of SBS-modified asphalt mixtures is 4.5%. Sasobit has no discernible impact on the optimum asphalt contents of asphalt mixtures [28]. Sasobit/SBS-modified asphalt was prepared by mixing Sasobit and SBS-modified asphalt, and then Sasobit/SBS-modified asphalt mixtures were prepared [29,30,31]. As stated in Table 4, two asphalt mixtures have the same AC-13 gradation, and the optimum asphalt content is 4.5%. The mixing temperatures of SBS-modified asphalt mixture and Sasobit/SBS-modified asphalt mixture were 180 °C and 160 °C, respectively, and the compaction temperatures were 170 °C and 150 °C, respectively [19]. The air void contents of the two asphalt mixtures after Marshall forming were tested. The results indicated that the air void contents of the two asphalt mixtures were basically the same, i.e., 4.2%.

### 2.3. Anti-Fuel Erosion Performance Tests of Asphalt

#### 2.3.1. Fuel Immersion Test 

**Figure 4:**
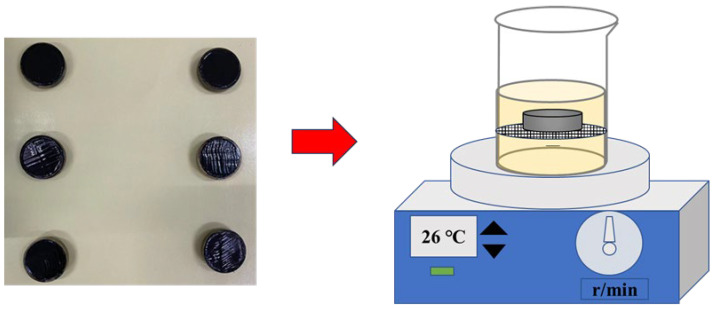
Fuel immersion experiment device of asphalt.

Giuliani and Merusi [15] confirm that the anti-fuel erosion ability of asphalt binder is correlated with the dissolution velocity of asphalt in fuel. Therefore, the influence of Sasobit on the anti-fuel of SBS-modified asphalt was assessed via the fuel immersion test, and the fuel immersion experiment device is shown in Figure 4. The copper ring used for the softening point test was used as a mold, and the asphalt was poured into the ring and cooled to perform the dissolution test at room temperature. The steps were as follows: a total of 250 mL fuel was added to a beaker and stirred at 100 rpm using a magnetic stirrer. The sample was placed on a metal mesh with a diameter of 0.5 mm and immersed in fuel. Every certain time, the mesh was taken out from the beaker, and the fuel on the surface of the sample was adsorbed by filter paper and weighed. For diesel, the selected time intervals were 5 min, 10 min, 20 min, 30 min, 60 min, 90 min and 120 min. Considering that the asphalt is highly soluble in gasoline, the selected time intervals were 5 min, 10 min, 20 min, 30 min. The samples of the diesel immersion were named D, and the samples of the gasoline immersion were named G.

The residual mass ratio of asphalt at fuel immersion time *t* is calculated according to Formula (1). *R_m_* decreases gradually with the increase in time, and *R_m_*(*t*) is fitted by function. The slope obtained by the fitting function is the dissolution velocity of asphalt in fuel.

(1) $$R_{\mathrm{mi}}=\frac{m_{i}}{m_{0}}\times 100\%$$

where *R_mi_* refers to the residual asphalt mass ratio at fuel immersion time *t*, and the unit is %; *m_i_* represents the mass of the sample at fuel immersion time *t*, and the unit is g; and *m*_0_ is the mass of the sample before fuel immersion, and the unit is g.

#### 2.3.2. Dynamic Shear Rheological (DSR) Test 

The DSR test was introduced to further investigate the influence of fuel erosion on the rheological properties of asphalt. The samples immersed in fuel were dried at room temperature for 12 h, and then the asphalt was recovered from the support (ring) and the DSR test was conducted. Phase angle and complex moduli were determined to be the evaluating index. With a 2 °C/min temperature rise, the test temperature ranged from 30 to 80 °C. Moreover, 10 rad/s was maintained as the constant frequency. 

### 2.4. Modification Mechanism Tests

#### 2.4.1. Fourier Transform Infrared Spectrometer (FT-IR) Test

The molecular structure and chemical composition of SBS-modified asphalt and Sasobit/SBS-modified asphalt were investigated via the FT-IR test, so as to determine the blending mode of Sasobit and SBS-modified asphalt. 

#### 2.4.2. Fluorescence Microscope Test

The microscopic morphology of SBS-modified asphalt and Sasobit/SBS-modified asphalt was observed via fluorescence microscope. The fluorescence image magnification was 400 times, and the influence of Sasobit on the phase structure of SBS-modified asphalt was investigated.

### 2.5. Anti-Fuel Erosion Performance Tests of Asphalt Mixtures 

#### 2.5.1. Fuel Immersion Test

**Figure 5:**
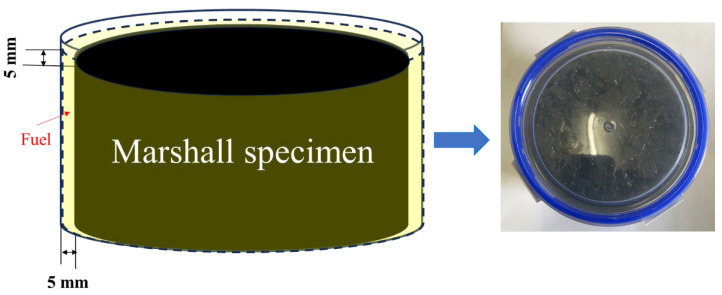
Fuel immersion experiment device of asphalt mixtures.

The Marshall specimens of asphalt mixtures were used as fuel-immersed samples, and the fuel immersion experiment device is represented in Figure 5. Considering that the amount of leakage fuel from the vehicle was generally not too large, and the fuel itself had a volatilization effect, the depth of fuel immersion for asphalt mixtures was determined to be 5 mm. And the lid of the container came with a sealing ring, the container was well sealed, and the fuel would not evaporate. The determination of the fuel immersion time is relatively critical. When the time is too short, the specimen has no obvious erosion phenomenon. When the time is too long, the specimen has serious peeling and shedding, and it is difficult to carry out subsequent performance tests. Thus, the times of diesel and gasoline immersion were set as 12 h, 24 h, 36 h and 48 h; the reasonable immersion times of diesel and gasoline were determined according to the fuel erosion of the specimen.

The fuel immersion test steps of the Marshall specimen were as follows: the Marshall specimen with a mass of *m*_0_ was placed in a container, the fuel was poured, and then the container was placed in a water bath of 26 °C. After the fuel immersion, the specimen was dried at the room temperature for 24 h, the residual fuel and aggregate particles on the surface of the specimen were cleaned, and the mass of the specimen was weighed *m*_1_. And the mass loss ratio after fuel immersion in formula (2) was used to appraise the anti-fuel erosion performances of asphalt mixtures. The mean Δ*m*_1_ value of triplicate samples was used for analysis.

(2) $${\Delta m}_{1}=\frac{m_{0}-m_{1}}{m_{0}}\times 100\%$$

where Δ*m*_1_ refers the mass loss ratio after the fuel immersion test, and the unit is %; *m*_0_ represents the mass of the sample before the fuel immersion test, and the unit is g; and *m*_1_ is the mass of the sample after the fuel immersion test, and the unit is g.

#### 2.5.2. Cantabro Test

The Cantabro test for asphalt mixtures is used to assess the degree of aggregate loss from the pavement under traffic loading due to insufficient adhesion. It is well known that the fuel damage affects the adhesion between aggregate and asphalt. Consequently, the Cantabro test can be selected to assess the influence of different fuels on the adhesion between asphalt and aggregate. However, it is worth noting that the immersion abrasion experiment is based on the use of water as the medium, as the sample is submerged in 60 °C water for 48 h, and the abrasion test is carried out after 24 h at room temperature. In this research, two kinds of fuel, diesel and gasoline, were used to replace water as the medium. The specimens were subjected to the abrasion test after the fuel immersion test. The mass loss ratio after the Cantabro test of the asphalt mixtures was calculated according to Formula (3), which was used as an index to assess the anti-fuel erosion performance. The mean Δ*m*_2_ value of triplicate samples was used for analysis.

(3) $${\Delta m}_{2}=\frac{m_{0}-m_{2}}{m_{0}}\times 100\%$$

where Δ*m*_2_ refers to the mass loss ratio after the Cantabro test, and the unit is %; *m*_0_ represents the mass of the sample before the fuel immersion test, and the unit is g; and *m*_2_ is the mass of the sample after the Cantabro test, and the unit is g.

#### 2.5.3. Marshall Stability (MS) Test

According to the Chinese standard JTG E20-2011 (T 0709-2011) [22], the Marshall stability test was conducted. The residual Marshall stability ratio after fuel immersion was calculated using Equation (4). The triplicate samples were tested, and the average value was analyzed.

(4) $$R_{\mathrm{MS}}=\frac{{MS}_{2}}{{MS}_{1}}\times 100\%$$

where *R_MS_* refers the residual Marshall stability ratio after fuel immersion, and the unit is %; *MS*_1_ is the Marshall stability of the specimen without fuel immersion, and the unit is kN; and *MS_2_* is the Marshall stability of the specimen after fuel immersion, and the unit is kN.

#### 2.5.4. Splitting Tensile Strength Test

The splitting tensile strength test was carried out according to the Chinese standard JTG E20-2011 (T 0716-2011), the test temperature was 15 °C and the loading rate was 50 mm/min. The residual splitting tensile strength ratio after fuel immersion was calculated using Equation (5). The triplicate samples were tested, and the average value was analyzed.

(5) $$R_{R_{T}}=\frac{R_{T1}}{R_{T0}}\times 100\%$$

where $R_{R_{T}}$ refers to the residual splitting tensile strength ratio after fuel immersion, and the unit is %; *R_T_*_0_ is the splitting tensile strength of the specimen without fuel immersion, and the unit is MPa; and *R_T_*_1_ is the splitting tensile strength of the specimen after fuel immersion, and the unit is MPa.

## 3. Results and Discussion

### 3.1. Influence of Sasobit on Anti-Fuel Erosion Performance of SBS-Modified Asphalt

#### 3.1.1. Residual Mass Ratio

**Figure 6:**
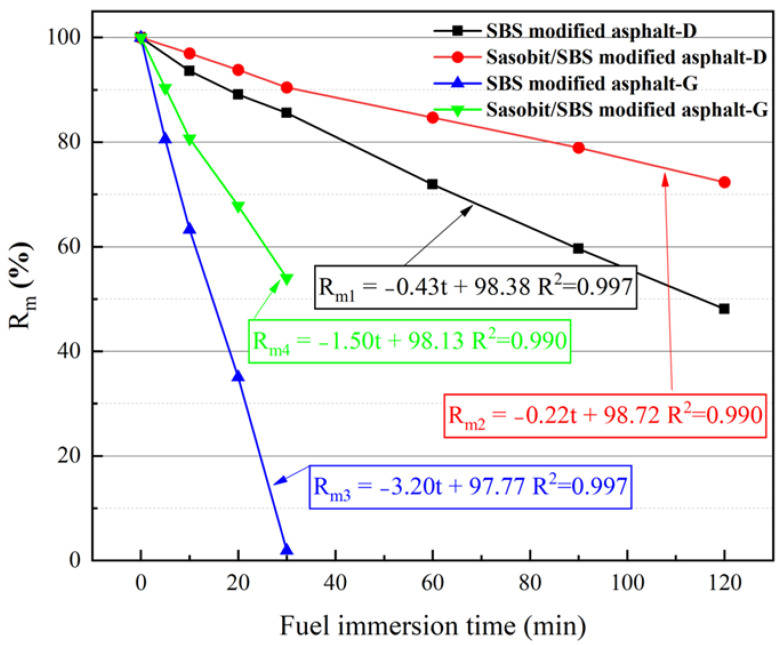
The influence of Sasobit on the *R_m_* of the SBS-modified asphalt immersed in diesel or gasoline.

The results of *R_m_* with the times of SBS-modified asphalt and Sasobit/SBS-modified asphalt in diesel and gasoline immersion are shown in Figure 6. It can be seen that *R_m_* decreases linearly with the fuel immersion time (t), and the linear fitting results of *R_m_*(*t*) are also demonstrated in Figure 6. The dissolution velocity of asphalt is expressed as the value of the slope of the fitted function. The dissolution velocities of SBS-modified asphalt in diesel and gasoline are 0.4%/min and 3.2%/min, respectively, which indicates that gasoline is far more harmful than diesel. The dissolution velocity of asphalt in fuel is larger, indicating that the anti-oil erosion ability of asphalt is worse. With the incorporation of Sasobit, the dissolution velocity of diesel-immersed SBS-modified asphalt reduces by 0.2%/min, and the dissolution velocity of the gasoline-immersed SBS-modified asphalt mixtures reduces by 1.7%/min. Therefore, the incorporation of Sasobit improves the anti-diesel and anti-gasoline erosion of SBS-modified asphalt, and the improvement in the anti-gasoline erosion is greater than that of anti-diesel erosion.

#### 3.1.2. Rutting Factor 

**Figure 7:**
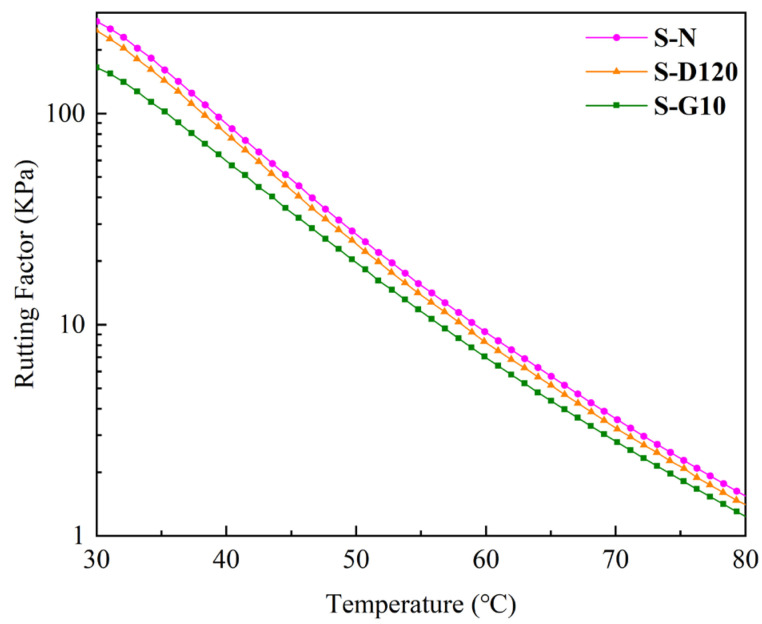
Influence of diesel and gasoline on the rutting factor of SBS-modified asphalt.

**Figure 8:**
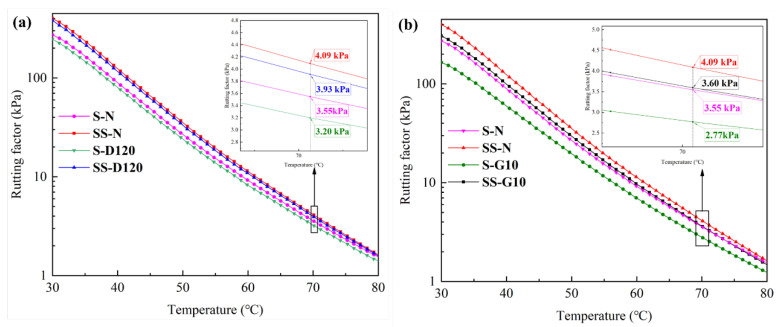
The influence of Sasobit on the rutting factor of SBS-modified asphalt immersed in fuel: (**a**) diesel; (**b**) gasoline.

The samples immersed in diesel for 120 min are tested via DSR. Due to the high solubility of the samples in gasoline, a large mass loss occurs after immersion in gasoline for 10 min; the DSR test is carried out on the samples immersed in gasoline for 10 min. The details of the six samples are represented in Table 5. The effects of diesel and gasoline erosion on the rutting factor (G*/sinδ) of SBS-modified asphalt are demonstrated in Figure 7, and the improvements caused by the influence of Sasobit on the rutting factor of SBS-modified asphalt after diesel and gasoline immersion are shown in Figure 8.

The rutting factor represents the high-temperature viscous component of the total binder strength. When the viscous component of asphalt is larger, the flow deformation at high temperatures is smaller, and the rutting resistance ability is stronger [32,33]. As illustrated in Figure 7, the rutting factors of S-D120 and S-G10 are lower than that of S-N, which shows that diesel and gasoline immersion reduces the rutting resistance ability of the SBS-modified asphalt, and the influence of gasoline immersion is greater than that of diesel immersion. It can be speculated that there is residual diesel in S-D120 and residual gasoline in S-G10. Since the asphalt is more soluble in gasoline than in diesel, more gasoline remains in the asphalt after gasoline immersion, which makes gasoline have a greater effect on rutting factor than diesel. What is more, with the increase in the test temperature, the residual diesel and gasoline in asphalt volatilize, and the difference between the rutting factor before and after fuel immersion decreases gradually. This is consistent with the conclusion of Felice et al. [34] that the softening effect of asphalt caused by fuel is related to temperature.

In order to quantitatively assess the improvement generated by Sasobit on the rutting factor of fuel erosion SBS-modified asphalt, the rutting factor at 70.1 °C is selected for analysis. As shown in Figure 8a, the rutting factor (70.1 °C) of SS-N is 0.5 kPa higher than that of S-N, so Sasobit improves the rutting resistance ability of SBS-modified asphalt [26]. After diesel immersion, the rutting factor (70.1 °C) of SBS-modified asphalt reduces by 0.4 kPa, and the rutting factor of Sasobit/SBS-modified asphalt reduces by 0.2 kPa. This shows that Sasobit reduces the diesel’s damage to the rutting resistance ability of SBS-modified asphalt. It can be seen in Figure 8b that, after gasoline immersion, the rutting factor (70.1 °C) of SBS-modified asphalt reduces by 0.8 kPa, and the rutting factor of Sasobit/SBS-modified asphalt reduces by 0.5 kPa. This shows that Sasobit reduces the gasoline’s damage to the rutting resistance ability of SBS-modified asphalt.

### 3.2. Anti-Fuel Erosion Mechanism of Sasobit

**Figure 9:**
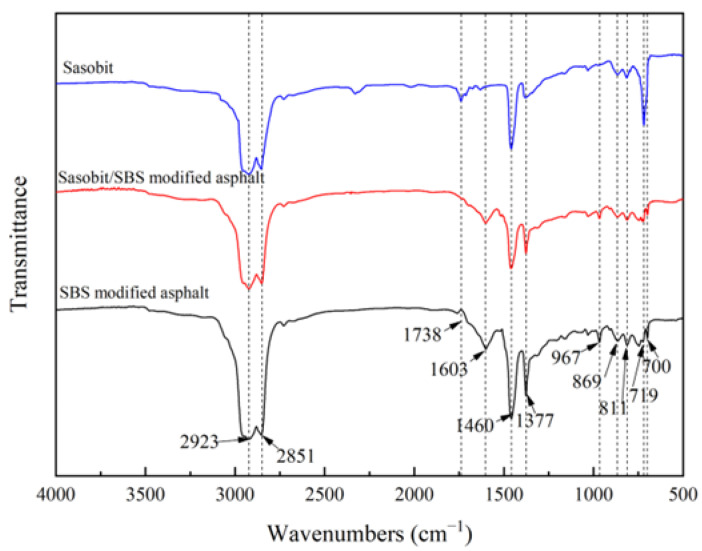
FT-IR spectra of Sasobit, SBS-modified asphalt and Sasobit/SBS-modified asphalt.

The FT-IR spectra of Sasobit, SBS-modified asphalt and Sasobit/SBS-modified asphalt are demonstrated in Figure 9. The FT-IR spectra of Sasobit show characteristic peaks at 2923 cm^−1^, 2851 cm^−1^, 1460 cm^−1^ and 719 cm^−1^ positions, indicating that Sasobit contains methylene. The characteristic peak at 1738 cm^−1^ position indicates that Sasobit contains the carbonyl group. The characteristic absorption peaks of SBS-modified asphalt and Sasobit/SBS-modified asphalt are located at approximately the same wavenumber positions. This shows that after the incorporation of Sasobit, the asphalt does not produce a new infrared absorption peak, there is no obvious chemical reaction, and the mixing method of Sasobit and SBS-modified asphalt is mainly physical blending. Specifically, the strongest peak of the two asphalts is 2923–2851 cm^−1^, which represents the stretching vibration of methylene. At 1603 cm^−1^, the main group causing the absorption infrared spectrum is the stretching vibration of the vinyl group. The C-H bending vibrations of methylene and methyl occur at 1460 cm^−1^ and 1377 cm^−1^, respectively. The characteristic peaks at 967 cm^−1^ and 700 cm^−1^ are caused by the characteristic functional groups of the polybutadiene and polystyrene of SBS, respectively. 

**Figure 10:**
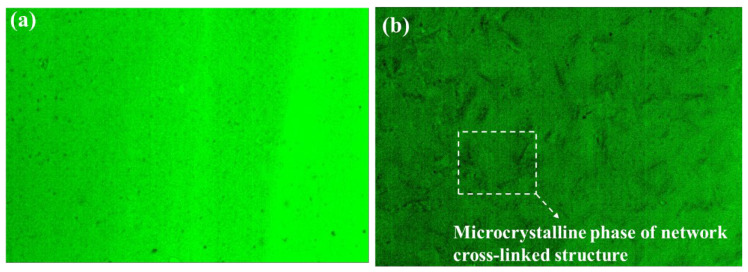
Fluorescence microscopic images of different asphalts: (**a**) SBS-modified asphalt; (**b**) Sasobit/SBS-modified asphalt.

The fluorescence microscopic images of SBS-modified asphalt and Sasobit/SBS-modified asphalt are represented in Figure 10. As illustrated in Figure 10a, the fluorescence microscopic image of SBS-modified asphalt shows a single green color, and the fluorescence of SBS is not obvious, indicating that asphalt and SBS are completely integrated. It can be seen in Figure 10b that after adding Sasobit to SBS-modified asphalt, there is a black network structure in the fluorescence microscopic image. This is due to the fact that when Sasobit is incorporated into hot SBS-modified asphalt, it can adsorb the saturated component similar to its structure in SBS-modified asphalt. When the temperature of SBS-modified asphalt decreases, Sasobit crystallizes and separates out with some saturated components, resulting in a continuous network lattice structure [35]. Filippo et al. [18] prove that there is a qualitative correspondence between the anti-fuel erosion of wax modified asphalt and the crystallinity of wax. Therefore, it can be speculated that the formed continuous lattice structure is equivalent to a protective film, which hinders fuel dissolution. Sasobit effectively improves the anti-fuel erosion performance of SBS-modified asphalt.

### 3.3. Effect of Fuel Immersion Time on the Mass Loss Ratio of SBS-Modified Asphalt Mixtures

**Figure 11:**
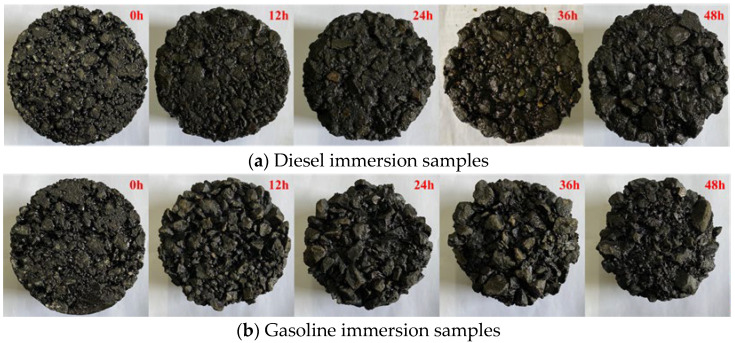
The appearance of the samples after the fuel immersion test.

**Figure 12:**
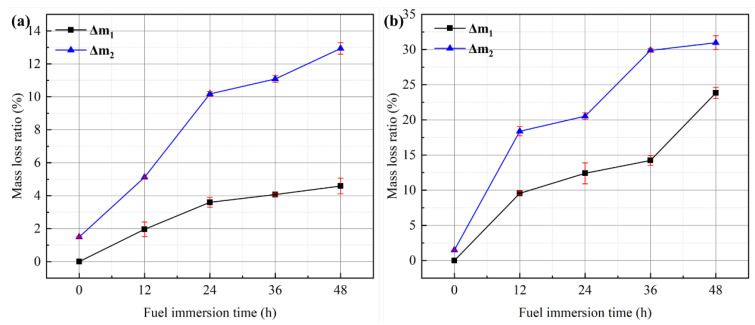
Mass loss ratios of SBS-modified asphalt mixtures with different fuel immersion times: (**a**) diesel immersion; (**b**) gasoline immersion.

The appearance of SBS-modified asphalt mixture samples after different times in the fuel immersion test is demonstrated in Figure 11. The mass loss ratio after the fuel immersion (Δ*m*_1_) and the mass loss ratio after the Cantabro test (Δ*m*_2_) of SBS-modified asphalt mixtures are demonstrated in Figure 12.

As shown in Figure 11, after immersion in diesel and gasoline, there are obvious asphalt film peeling and aggregate shedding on the surfaces of the samples, and as the immersion time increases, the damage to the samples becomes more and more serious. The integrity of the specimen is still basically maintained after 48 h of diesel immersion, but the integrity of the specimen is destroyed after more than 24 h of gasoline immersion, indicating that the harm of gasoline erosion is much greater than that of diesel erosion.

As can be seen in Figure 12a, the growth velocity of Δ*m*_1_-D slows down after 24 h, which is consistent with the research of Li et al. [5]. This is due to the fact that SBS forms a continuous three-dimensional spatial network structure in the asphalt to attenuate the erosion of diesel. It is evident that SBS-modified asphalt mixtures have certain anti-diesel abilities. The change trend of Δ*m*_2_-D and Δ*m*_1_-D with time is roughly the same, but in the 12–24 h and 36–48 h intervals, the growth velocity of Δ*m*_2_-D accelerates, while Δ*m*_1_-D does not show this trend. As can be seen in Figure 12b, Δ*m*_1_-G is much larger than Δ*m*_1_-D. The trends of Δ*m*_1_-G and Δ*m*_1_-D are similar before 36 h, but the growth velocity of Δ*m*_1_-G becomes faster after 36 h. This is because the solubility of gasoline is greater than that of diesel, so the protective film is destroyed earlier. The change trends of Δ*m*_2_-G and Δ*m*_1_-G with time are roughly the same in the 0–24 h interval, but the gap is very large in the 24–48 h interval.

Compared with Δ*m*_1_, the calculation of Δ*m*_2_ takes into account the mass losses of the fuel immersion test and the abrasion test: Δ*m*_2_ can more accurately reflect the influence of fuel damage on asphalt mixtures, which can be used as an assessment index for the anti-fuel ability of asphalt mixtures. The growth velocity of Δ*m*_2_-D tends to be gentle in the range of 24–48 h, and the growth velocity of Δ*m*_2_-G tends to be gentle in the ranges of 12–24 h and 36–48 h. In order to assess the anti-fuel performances of asphalt mixtures, the diesel immersion test time is determined to be 24 h. The gasoline immersion test time is determined to be 12 h or 36 h, but considering that the integrity of the specimen is destroyed after gasoline immersion for more than 24 h, the gasoline immersion test time is finally determined to be 12 h. This immersion time parameter does not affect the subsequent performance tests of asphalt mixtures and can quickly simulate the damage caused by fuel to asphalt mixtures. 

### 3.4. Effect of Sasobit on Anti-Fuel Erosion Abilities of SBS-Modified Asphalt Mixtures

According to the change rule of the mass loss ratios of SBS-modified asphalt mixtures with fuel immersion time, SBS-modified asphalt mixtures and Sasobit/SBS-modified asphalt mixtures are subjected to a diesel immersion test for 24 h or a gasoline immersion test for 12 h, followed by abrasion, MS or split tensile strength tests, respectively. The samples without fuel immersion are named N, the samples immersed in diesel for 24 h are named D, and the samples immersed in gasoline for 12 h are named G. The influence of Sasobit on the anti-fuel erosion abilities of SBS asphalt mixtures is investigated based on the mass loss ratio and mechanical properties.

#### 3.4.1. Mass Loss Ratio

**Figure 13:**
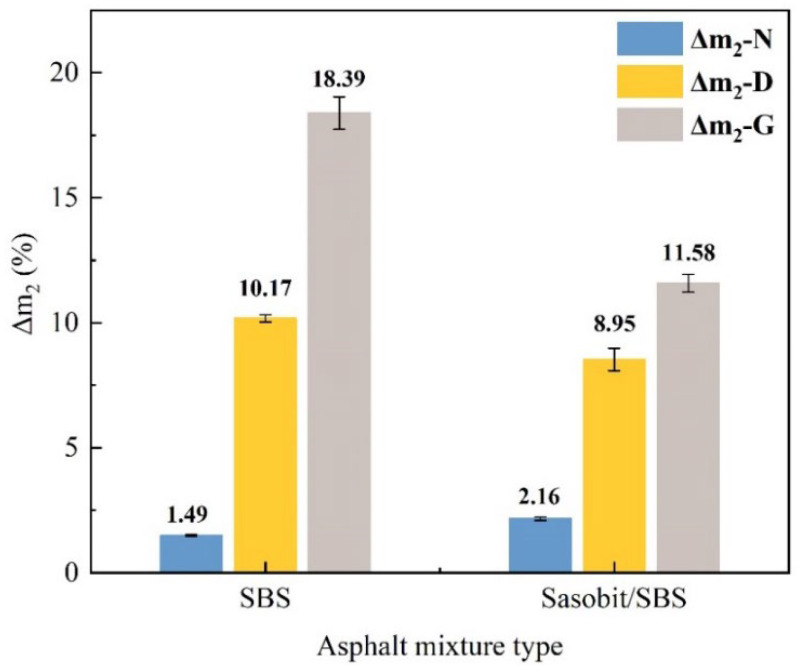
The influence of Sasobit on the Δ*m*_2_ of SBS-modified asphalt mixtures.

The mass loss ratio after the Cantabro test (Δ*m*_2_) is represented in Figure 13. The larger Δ*m*_2_ indicates that the spalling of the specimen is serious, and the adhesion between asphalt and aggregate is poor.

It can be seen in Figure 13 that the incorporation of Sasobit increases the Δ*m*_2_ of SBS-modified asphalt mixtures without fuel immersion by 0.7%, demonstrating that Sasobit will slightly reduce the adhesion of SBS-modified asphalt and aggregates [27]. With the incorporation of Sasobit, the Δ*m*_2_ of diesel-eroded SBS-modified asphalt mixtures reduces by 1.2%, and the Δ*m*_2_ of gasoline-eroded SBS-modified asphalt mixtures reduces by 6.8%. This indicates that Sasobit mitigates the damage caused by fuel erosion to the adhesion performances of SBS-modified asphalt and aggregates and improves the anti-gasoline erosion of SBS-modified asphalt mixtures more than the anti-diesel erosion of SBS-modified asphalt mixtures.

#### 3.4.2. Mechanical Properties

**Figure 14:**
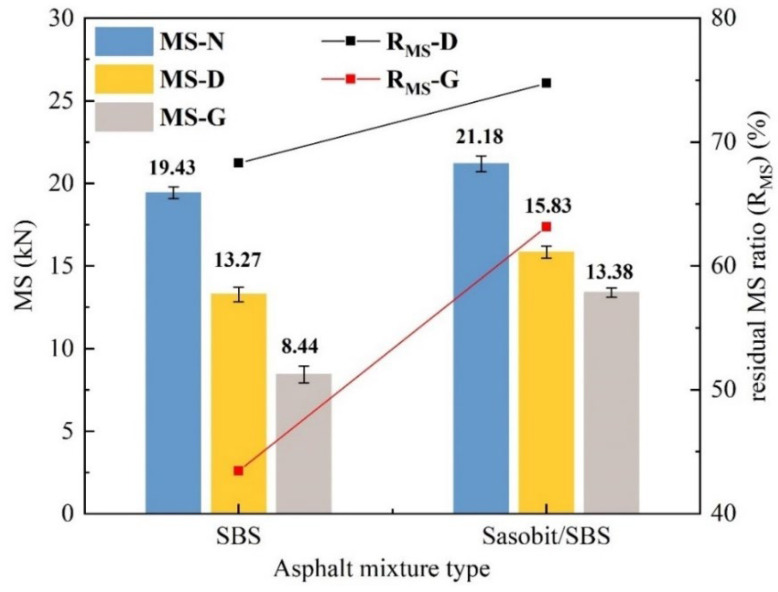
The influence of Sasobit on the *MS* of SBS-modified asphalt mixtures.

The Marshall stability (*MS*) and the residual Marshall stability ratio after fuel immersion (*R_MS_*) are shown in Figure 14, and the *MS* value can reflect the high-temperature properties of asphalt mixtures; the larger *R_MS_* indicates that the damage caused by fuel erosion to the high-temperature properties of asphalt mixtures is smaller. With the incorporation of Sasobit, the *R_MS_* of diesel-eroded SBS-modified asphalt mixtures increases by 6.4%, and the *R_MS_* of gasoline-eroded SBS-modified asphalt mixtures increases by 19.7%. Therefore, the incorporation of Sasobit weakens the damage caused by diesel and gasoline to the high-temperature properties of SBS-modified asphalt mixtures. 

**Figure 15:**
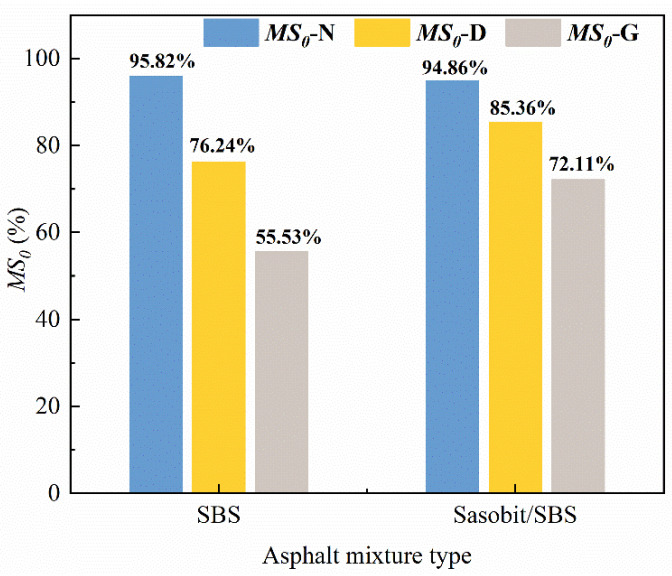
The influence of Sasobit on the *MS*_0_ of the SBS-modified asphalt mixtures.

The water immersion residual stability (*MS*_0_) is represented in Figure 15, and the *MS_0_* is used as an index to assess the moisture susceptibility; the larger *MS_0_* shows that the moisture susceptibility properties of asphalt mixtures are better. With the incorporation of Sasobit, the *MS*_0_ of diesel-eroded SBS-modified asphalt mixtures increases by 9.1%, and the *MS_0_* of gasoline-eroded SBS-modified asphalt mixtures increases by 16.6%. Therefore, Sasobit attenuates the damage caused by diesel and gasoline erosion to the moisture susceptibility levels of SBS-modified asphalt mixtures.

**Figure 16:**
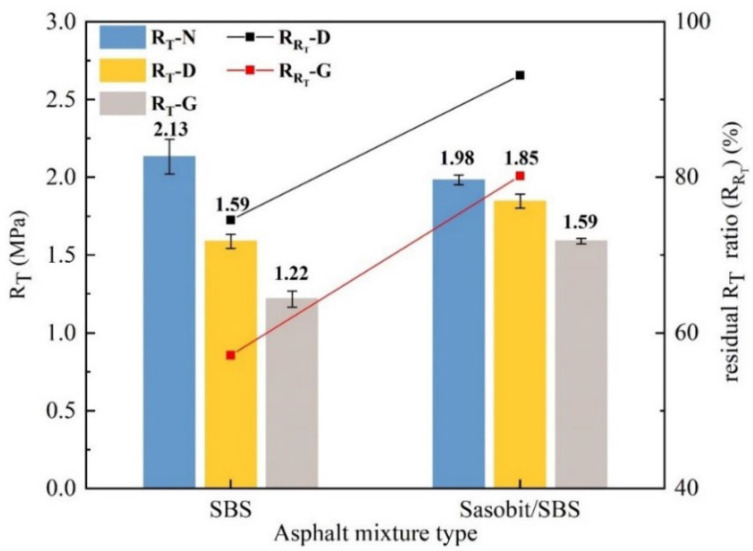
Influence of Sasobit on the *R_T_* of SBS-modified asphalt mixtures.

The splitting tensile strength (*R_T_*) and the residual splitting tensile strength ratios after fuel immersion ($R_{R_{T}}$) are demonstrated in Figure 16, where *R_T_* is used as an index to assess the fracture performance; a larger $R_{R_{T}}$ indicates that the damage caused by fuel to the crack resistance of asphalt mixtures is smaller. With the incorporation of Sasobit, the $R_{R_{T}}$ of diesel-eroded SBS-modified asphalt mixtures increases by 18.6%, and the $R_{R_{T}}$ of gasoline-eroded SBS-modified asphalt mixtures increases by 23.1%. This suggests that Sasobit weakens the damage caused by diesel or gasoline to the fracture properties of SBS-modified asphalt mixtures. 

**Figure 17:**
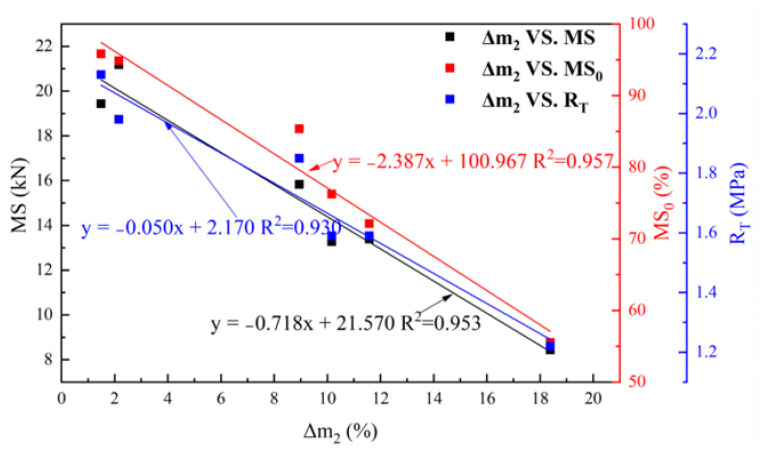
Correlations of different anti-fuel erosion tests results.

The correlation coefficients between the Cantabro test results and the mechanical properties test results are shown in Figure 17. As can be observed, the determination coefficient R^2^ values of Δ*m*_2_ and *MS*, *MS*_0_ and *R_T_* are all greater than 0.9, indicating that the Δ*m*_2_ is reasonable and accurate as an index to assess the anti-fuel performances of asphalt mixtures.

## 4. Conclusions

In this research, Sasobit is used as an anti-fuel erosion agent to explore its effect on the anti-fuel ability of SBS-modified asphalt and its mixtures. The following findings are reached in light of the analysis above:

- The dissolution velocity of Sasobit/SBS-modified asphalt is 0.2%/min for diesel erosion, while it is 1.7%/min for gasoline erosion, lower than control sample without Sasobit. Diesel and gasoline erosion significantly reduce the rutting resistance ability of SBS-modified asphalt, while Sasobit weakens the damage caused by diesel and gasoline to the rutting resistance ability of SBS-modified asphalt. Therefore, Sasobit improves the anti-diesel ability of SBS-modified asphalt and significantly improves the anti-gasoline ability of SBS-modified asphalt.
- The major method for incorporating Sasobit into SBS-modified asphalt is physical blending. The continuous network crystal structure formed by Sasobit in SBS-modified asphalt effectively improves the anti-fuel ability of SBS-modified asphalt and its mixtures.
- According to the mass loss ratio after fuel immersion and the mass loss ratio after the Cantabro test of SBS-modified asphalt mixtures with different fuel immersion times, the mass loss ratio after the Cantabro test can more accurately reflect the influence of fuel erosion on asphalt mixtures.
- The mass loss ratio after the Cantabro test of Sasobit/SBS-modified asphalt mixtures is 1.2% for diesel erosion, while it is 6.8% for gasoline erosion, lower than control sample without Sasobit. Sasobit weakens the damage caused by diesel and gasoline to the high-temperature performance, moisture susceptibility and fracture properties of SBS-modified asphalt mixtures. Therefore, Sasobit significantly improves the anti-diesel and anti-gasoline performances of SBS-modified asphalt mixtures. It is reasonable and accurate to use the mass loss ratio after the Cantabro test as an index to evaluate the anti-fuel erosion abilities of asphalt mixtures.

## Figures and Tables

**Table 1:** The basic properties of the SBS-modified asphalt used in this research.

Properties	Standard Value	Measured Value	Test Methods
Penetration (25 °C, 0.1 mm)	40–60	56.7	T0604-2011
Viscosity (135 °C, Pa·s)	≥0.35	1.13	T0625-2011
Softening point (°C)	≥60	76.1	T0606-2011
Ductility (5 °C, cm)	≥20	59.0	T0605-2011
Density (g/cm^3^)	-	1.04	T0603-2011

**Table 2:** The basic properties of Sasobit used in this research.

Properties	Melting Point (°C)	Flash Point (°C)	Viscosity (135 °C, Pa·s)	Penetration (25 °C, 0.1 mm)
Measured value	100	290	5.47 * 10^−3^	1

**Table 3:** The main properties of Sasobit/SBS-modified asphalt.

Properties	Measured Value	Test Methods
Penetration (25 °C, 0.1 mm)	38.1	T0604-2011
Softening point (°C)	89.3	T0606-2011
Ductility (5 °C, cm)	40.0	T0605-2011
Viscosity (135 °C, Pa·s)	0.99	T0625-2011

**Table 4:** The technical indexes of two asphalt mixtures.

Asphalt	Optimal Asphalt Content (%)	Mixing Temperature (°C)	Compaction Temperature (°C)	Relative Density of Bulk Volume	Maximum Theoretical Relative Density	Air Void Content (%)
SBS-modified asphalt	4.5	180	170	2.628	2.744	4.23
Sasobit/SBS-modified asphalt	4.5	160	150	2.626	2.741	4.20

**Table 5:** Sample information of DSR test.

Description of Sample	Asphalt	Fuel Type	Fuel Immersion Time (min)
S-N	SBS-modified asphalt	-	-
S-D120	SBS-modified asphalt	diesel	120
S-G10	SBS-modified asphalt	gasoline	10
SS-N	Sasobit/SBS-modified asphalt	-	-
SS-D120	Sasobit/SBS-modified asphalt	diesel	120
SS-G10	Sasobit/SBS-modified asphalt	gasoline	10

## Data Availability

The raw/processed data required to reproduce these findings cannot be shared at this time as the data also form part of an ongoing study.
